# A Three-Dimensional Melamine Sponge Modified with MnOx Mixed Graphitic Carbon Nitride for Photothermal Catalysis of Formaldehyde

**DOI:** 10.3390/molecules27165216

**Published:** 2022-08-16

**Authors:** Rongyang Yin, Pengfei Sun, Lujun Cheng, Tingting Liu, Baocheng Zhou, Xiaoping Dong

**Affiliations:** Department of Chemistry, Key Laboratory of Surface & Interface Science of Polymer Materials of Zhejiang Province, Zhejiang Sci-Tech University, 928 Second Avenue, Xiasha Higher Education Zone, Hangzhou 310018, China

**Keywords:** photothermal conversion, graphitic carbon nitride, oxygen vacancy, manganese oxide, formaldehyde

## Abstract

Much attention has been paid to developing effective visible light catalytic technologies for VOC oxidation without requiring extra energy. In this paper, a series of sponge-based catalysts with rich three-dimensional porosity are synthesized by combining MnOx and graphitic carbon nitride (GCN) with commercial melamine sponges (MS) coated with polydopamine (PDA), demonstrating excellent photothermal catalytic performance for formaldehyde (HCHO). The three-dimensional porous framework of MS can provide a good surface for material modification and a reliable interface for gas-solid interaction. The grown layer of PDA framework not only increases the near-infrared wavelength absorption for improving the light-to-heat conversion of catalysts, but also brings excellent adhesion for the subsequent addition of MnO_X_ and GCN. The efficient formaldehyde oxidation is attributed to the sufficient oxygen vacancies generated by co-loaded MnO_X_ and GCN, which is conducive to the activation of more O^2−^ in the oxidation process. As the surface temperature of catalyst rapidly increases to its maximum value at ca. 115 °C under visible light irradiation, the HCHO concentration drops from 160 ppm to 46 ppm within 20 min. The reaction mechanism is certified as a classical Mars-van Krevelen mechanism based on the photo-induced thermal catalysis process.

## 1. Introduction

HCHO has been extensively found in boards, paints, carpets, and wallpapers commonly used in interior decoration. According to the International Agency for Research on Cancer, formaldehyde can cause cancer in humans, especially nasopharyngeal cancer and leukemia [1,2]. With the general public’s need for health and concern for a more efficient use of resources, it is of great importance to develop efficient and clean indoor HCHO degradation methods. At present, HCHO treatment methods mainly include absorption [3], biological incineration [4] and catalytic oxidation [5]. Among them, the activated carbon adsorption method is the most widely used; however, this requires regularly replacing activated carbon and recovering activated carbon wastes, thus resulting in higher elution costs. In contrast, catalytic oxidation converts exhaust gas into harmless carbon dioxide and water, which is more suitable for HCHO purification. In catalytic oxidation, synergistic photothermal catalysis technology is considered as an efficient and low-carbon HCHO removal technology that integrates the advantages of both photocatalysis and thermocatalysis. Under illumination, the material with photothermal effect does not directly change the state of the internal electrons after absorbing photons, but converts light energy into lattice thermal vibration, thus increasing the material’s temperature. The catalysts can be activated using natural light sources without requiring additional energy.

Manganese oxide (MnOx), as a common transition metal oxide, has the above photothermal properties. Li Yuanzhi et al. reported MnOx nano-catalysts with various structures that have been proven to activate lattice oxygen with sunlight and efficiently remove various VOCs [6]. Wang Wenzhong also modified MnOx with cobalt oxide with significantly improved photothermal catalytic performance for the oxidation of VOCs, which was attributed to the changing lattice oxygen structure in MnOx [7]. Wang Zhongsen also synthesized two-dimensional nanosheets of MnOx by adding graphene oxide and polymeric carbon nitride, achieving excellent visible light oxidation efficiency of HCHO [2]. The photothermal effect of MnOx mainly originates from the non-radiative recombination of electron-hole pairs produced by the d-d transition of metal ions upon the absorption of photons. The surface temperature of MnOx increases even more efficiently after being modified by other photothermal materials such as black carbon or graphene, as black carbon-based materials are able to more sufficiently absorb the solar spectrum than metal oxide, ranging from ultraviolet light to the entire near-infrared band [8]. Thus, these materials endow MnOx with a stronger light-to-heat conversion ability, eventually inducing a thermal catalysis process with the Mars-van Krevelen (MvK) mechanism [9,10].

A three-dimensional porous composite MS/PDA/MnOx/GCN catalyst is synthesized by a simple method in this paper. A melamine sponge (MS) is used as a three-dimensional porous framework where a layer of polydopamine (PDA) is grown. Similar to carbon-based materials, black PDA has excellent light-to-heat conversion properties due to its strong ability to absorb near-infrared wavelengths [11]. Additionally, PDA exhibits excellent adhesion, which can firmly adhere subsequent MnOx nanosheets and graphitic carbon nitride (GCN) to further improve oxidation susceptibility and light-to-heat conversion. Compared with other photocatalysts without a fixed structure, this 3D skeleton structure exhibits better photoresponse performance. It provides a larger surface area for active phase loading and visible light absorption, which thus improves the removal efficiency of HCHO.

According to the experimental results, the synthesized catalyst exhibits a considerable HCHO photothermal catalytic removal efficiency with HCHO concentration dropping from 160 ppm to 46 ppm in 20 min. The morphology and surface elemental properties of the catalyst are also studied by SEM, HR-TEM, XRD, XPS, ESR, etc. Moreover, a surface reaction mechanism is proposed by using in situ DRIFTS and reactive oxygen species detection.

## 2. Experimental

### 2.1. Catalyst Synthesis

**Figure 1:**
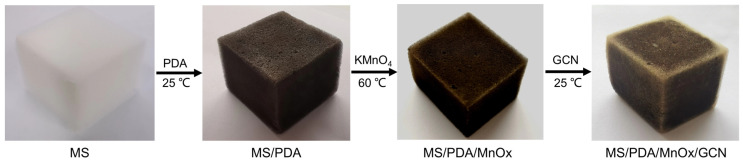
Schematic diagram of the sample preparation process.

The flow chart of the synthesis process is shown in Figure 1: 50 mg dopamine hydrochloride was dispersed into 50 mL Tris-HCl solution (pH = 8.5) under agitation. Then, a blank melamine sponge (MS) with size = 2.5 cm^3^ was immersed into the solution and stirred magnetically for 6 h, which was named MS/PDA. After being washed with distilled water, the MS/PDA was further immersed in 50 mL 0.05 mol/L KMnO_4_ solution for 5 h at 60 °C and dried at 60 °C. The prepared sample was named MS/PDA/MnOx. Finally, the dried MS/PDA/MnOx was immersed into 0.2 g/L GCN suspension for 10 min by ultrasound. The obtained sample was washed by distilled water and dried at room temperature, which was named MS/PDA/MnOx/GCN. In order to investigate the influence of the loading sequence between GCN and MnOx on photothermal response performance, we also prepared the MS/PDA/GCN/MnOx catalyst by exchanging the synthesis order of the last two steps.

### 2.2. Characterization

The catalyst was characterized by scanning electron microscopy (SEM) using a field emission scanning electron microscope (Model SU-8100, Hitachi Co., Tokyo, Japan) equipped with an energy dispersive X-ray spectrometer (EDS). X-ray diffraction (XRD) was tested by a powder diffractometer with Cu *Kα* radiation (Model D/max RA, Rigaku Co., Tokyo, Japan). The chemical compositions and states were explored by X-ray photoelectron spectroscopy (XPS, Model AXIS UltraDLD, Kratos Co., Manchester, UK) using an Al *Kα* monochromatic source. Electron spin resonance (ESR) spectra were tested using an electron paramagnetic resonance spectrometer (Model ESP 300 E, Bruker Co., Ettlingen, Germany) equipped with a xenon lamp as a light source (420–600 nm). UV-vis-NIR diffuse-reflectance spectra (DRS) were analyzed on a HITACHI U-4100 spectrophotometer. The changes in the surface temperature of different catalysts were characterized by a thermal image (Model H21PRO, Hikvision Co., Hangzhou, China).

### 2.3. Photothermal Catalytic Performance

The performance of the catalyst was tested in a 0.5 L glass container containing a 0.2 g sponge sample, with observation glass in the middle. After the gas-phase reactor was preheated to ca. 40 °C, 2.0 μL HCHO (37%) solution was injected into the gas-phase reactor till completely evaporated. At this time, the initial concentration of HCHO was approximately ca. 160 ppm. A 300 W xenon lamp (Model HSX-F300, NBet Co., Beijing, China) was used as light source. The HCHO removal activity under light irradiation was indirectly evaluated by measuring the absorbance obtained by phenol reagent spectrophotometry (Model UV-2600, Shimadzu Co., Kyoto, Japan). During measurements, 5 mL gas was sampled with a syringe and injected into 5 mL 0.05 mol/L phenol reagent (MBTH hydrochloride hydrate) solution. Subsequently, 0.4 mL 1 wt.% NH_4_Fe(SO_4_)_2_ solution was added. After approximately 10 min, the absorbance of the above solution at 630 nm was measured. The changing surface temperature of the catalyst under light illuminations was also recorded by an infrared thermometer in the experimental process (Model H16, Hikvision Co., Hangzhou, China). A picture of the experimental set-up has been listed in the Supplementary Material.

### 2.4. Reaction Mechanism

The HCHO decomposition mechanism was clarified based on in situ diffuse reflectance infrared Fourier transform spectroscopy (in situ DRIFTS) using a Bruker Co. Model TENSOR II spectrometer. The reaction spectra of HCHO on the surface of the catalyst were acquired by accumulating 32 scans at a 4 cm^−1^ resolution under the irradiation of a xenon lamp. To confirm the formation of oxidized OH radical and O^2^^−^ radical during the reaction, the photoluminescence (PL) method with terephthalic acid (TA) oxidized by ·OH and blue tetrazolium (NBT) by O^2^^−^ was employed. Both reaction products were detected by changing the absorbance using a fluoroanalyzer (Model Fluoromax-4, HORIBA Co., Kyoto, Japan).

## 3. Results and Discussion

### 3.1. Photothermal Catalytic Performance of Formaldehyde

**Figure 2:**
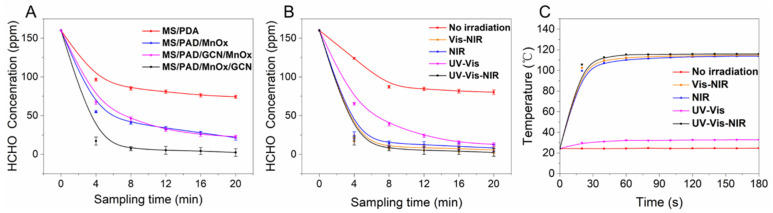
(**A**) Catalytic performance for HCHO degradation under the UV-Vis-NIR irradiation; (**B**) catalytic performance of catalyst MS/PDA/MnOx/GCN for HCHO degradation under irradiation with different wavelength ranges; (**C**) temperature change for catalyst MS/PDA/MnOx/GCN under irradiation with different wavelength ranges.

The photothermal activity of different sponge loaded catalysts was evaluated by HCHO degradation (the concentration of HCHO was ca. 160 ppm) using 300 W xenon lamps. As displayed in Figure 2A, the HCHO concentration of four catalysts decreased to varying degrees. For MS/PDA, the poor removal efficiency of HCHO was obviously due to the lack of reactive oxygen species caused by the absence of loaded metal oxide. The catalysts MS/PDA/MnOx and MS/PDA/GCN/MnOx exhibited similar HCHO removal efficiency. The HCHO concentration declined from 160 ppm to 46 ppm within 20 min, implying that neither the oxidation susceptibility of subsequently loaded MnOx nor the photothermal synergistic performance of the catalyst was improved by adding GCN. However, the catalyst MS/PDA/MnOx/GCN revealed significantly improved HCHO removal performance compared with MS/PDA/GCN/MnOx, suggesting that the loading sequence of GCN had a great effect on the performance of the photothermal co-catalytic system.

Figure 2B shows the degradation of HCHO under different light sources. Obviously, the degradation efficiency of HCHO without any light irradiation was not ideal. Compared with the rapid decline in the degradation efficiency of HCHO under other conditions, NIR light played a key role in the HCHO removal though UV and visible light also contributed to the photodegradation of HCHO. Figure 2C also exhibits the change in the surface temperature of the catalyst MS/PDA/MnOx/GCN under the condition of different light sources. The surface temperature of the catalyst MS/PDA/MnOx/GCN filtered by NIR light did not obviously change compared with that with the presence of NIR light. The latter temperatures all significantly increased to ca. 115 °C. Thus, we believe the degradation of HCHO on the manganese-based catalyst was mainly affected by increased temperature, which induced the well-known Mars-van Krevelen process that decomposes C_x_H_y_ utilizing·O^2^^−^, OH and other oxidation groups [10,12].

The UV-Vis-NIR DRS was also tested to evaluate the photo-thermal effect of four different catalysts. As shown in Figure S1, four catalysts all exhibited considerable NIR absorption (wavelength ranging from 400 to 800 nm) due to the loaded PDA. These were the efficient NIR-absorbing materials as reported in the literature [11,13]. For MS/PDA/MnOx/GCN, the highest NIR absorption was found. The NIR absorption of the catalyst MS/PDA/MnOx/GCN was found to be the highest because its outermost surface was further wrapped by GCN, which further increased NIR light absorption [14]. The changes in the surface temperature of the four catalysts were tested by thermal imaging, as exhibited in Figures S2 and S3. This was consistent with the UV-Vis-NIR DRS result that the catalyst MS/PDA/MnOx/GCN displayed the highest temperature.

### 3.2. Morphological Characteristics

**Figure 3:**
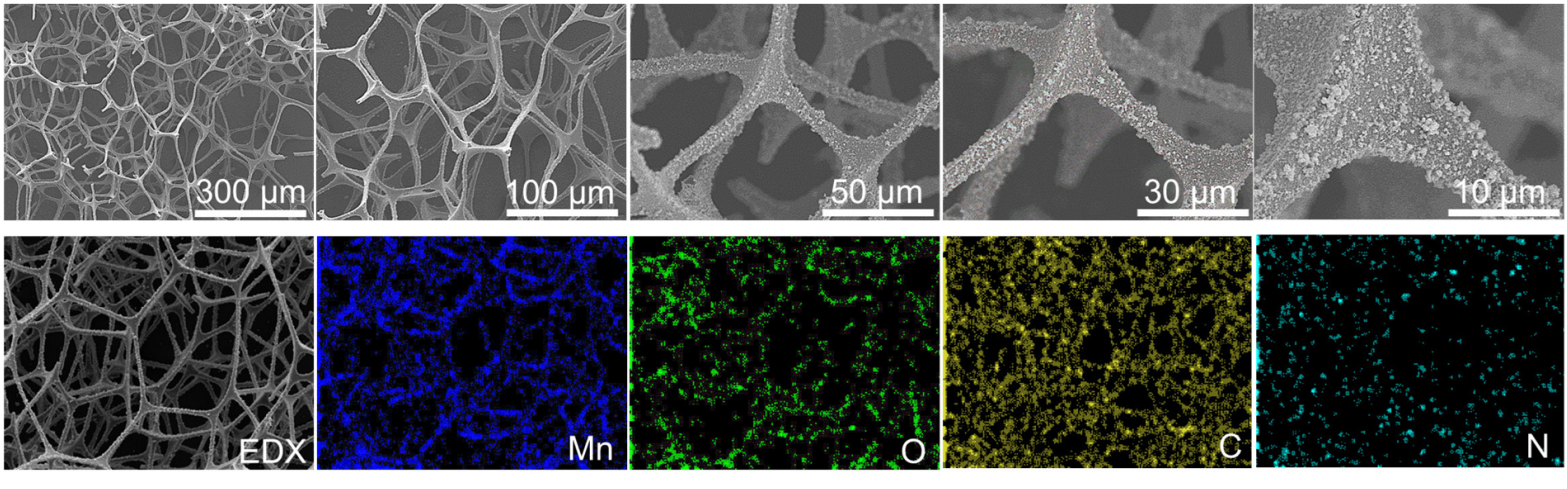
SEM image and EDX mapping of the catalyst MS/PDA/MnOx/GCN.

**Figure 4:**
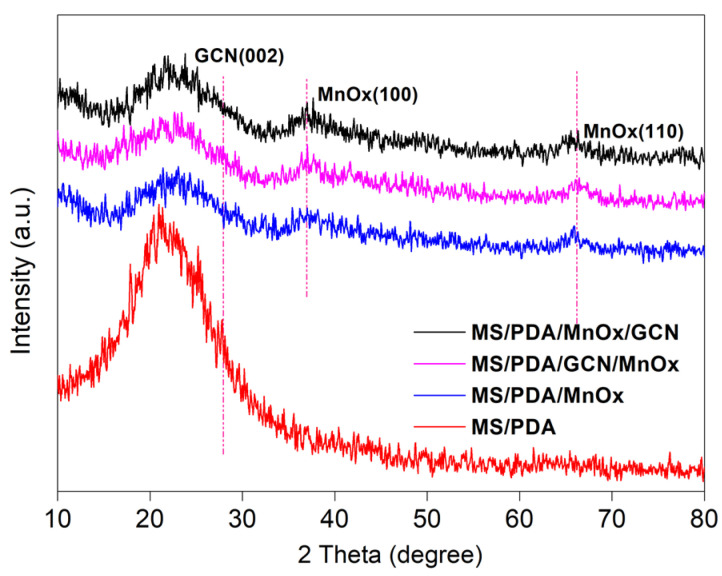
XRD profiles of the catalysts MS/PDA, MS/PDA/MnOx, MS/PDA/GCN/MnOx and MS/PDA/MnOx/GCN.

As shown in Figure 3, the SEM image of the catalyst MS/PDA/MnOx/GCN exhibited uniformly attached MnOx on the 3D structure of MS. The EDX mapping also confirmed that the scanning of the Mn and O elements was in accordance with the MS skeleton. The XRD result is displayed in Figure 4. It can be observed from the figure that all the samples revealed an obvious peak at 2θ = ca. 22°, which was ascribed to the surface diffraction of the melamine sponge [11,13]. With the increase in surface load, the intensity of the diffraction peak became weaker. The characteristic peaks of Mn loaded samples at 2θ = 36.5° and 65.5° were ascribed to the (100) and (110) planes of an Akhtenskite MnOx phase (JCPDS NO.30-0820) [11,13], indicating that MnOx was effectively formed on the surface of the melamine sponge merely by using potassium permanganate. However, the loaded PDA and GCN were not identified by XRD diffraction patterns mainly due to the fact that the loading of these two substances always presented good dispersion, as exhibited by the SEM image.

### 3.3. Surface Properties

**Figure 5:**
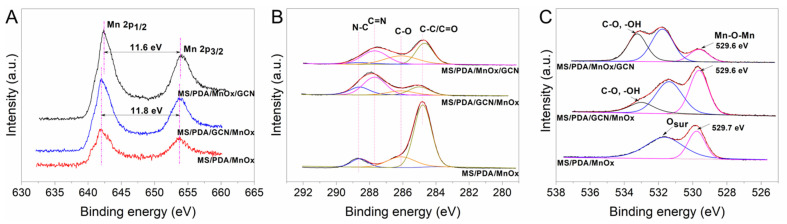
XPS spectra of the catalysts MS/PDA/MnOx, MS/PDA/GCN/MnOx and MS/PDA/MnOx/GCN: (**A**) Mn 2p; (**B**) C 1s; (**C**) O 1s.

In order to further understand the different surface properties of the catalysts MS/PDA/MnOx, MS/PDA/MnOx/GCN and MS/PDA/GCN/MnOx, XPS analysis was carried out. As shown in Figure 5A, Mn 2p spectrum showed two characteristic peaks ascribed to Mn 2p 3/2 (641.9 eV) and Mn 2p 1/2 (653.4 eV) [15,16], which suggests the presence of MnO_2_. It should be noted that the splitting energy between these two characteristic peaks was 11.6 eV for MS/PDA/MnOx/GCN and 11.8 eV for MS/PDA/MnOx and MS/PDA/GCN/MnOx. The splitting energy of MS/PDA/MnOx/GCN decreased because of the enhanced π electron cloud density [16], which was probably originated from the reaction between the residual GCN on the external surface of MnOx and its functional groups.

The C 1s spectrum (Figure 5B) shows four peaks at 284.4, 286.2, 287.7 and 288.6 eV, respectively. The peak at 287.7 eV was ascribed to carbon in the melamine sponge conjugated system [11]. The peaks at 284.4 and 286.2 eV corresponded to sp2 graphite carbon in the PDA structure and C-O carbon in the benzene ring of the PDA molecules, respectively [11,17]. Additionally, the peak at 287.7 eV was ascribed to C=N to the GCN structure [18]. It was also observed that the order of GCN addition determined whether GCN reacted with PDA or MnOx. For MS/PDA/GCN/MnOx, PDA (peak at 284.4 eV) was found to be depleted compared with MS/PDA/MnOx/GCN and MS/PDA/MnOx. For the latter two catalysts, the PDA firstly reacted with manganese, resulting in a similar PDA structure. Especially for MS/PDA/MnOx/GCN, as GCN was loaded after MnOx, a tight bonding with MnOx as the splitting energy of Mn 2p shift was detected, as shown in Figure 5A.

The O 1s spectrum (Figure 5C) was divided into three peaks. The peak at 529.6 eV was ascribed to the lattice oxygen of Mn-O-Mn in MnOx [19], that at 531.3 eV was ascribed to surface chemisorbed oxygen species [20], and that at 532.8 eV was ascribed to hydroxyl groups such as Mn-OH or H-OH [21,22]. All of three Mn loaded catalysts exhibited chemisorbed oxygen species and lattice oxygen due to the predominant oxygen species formed on the surface MnOx. After the addition of GCN, the ionized oxygen species such as OH^-^ emerged, indicating that the functional groups of GCN promoted the transformation of Mn-O to Mn-OH. As reported in the literature [23,24,25], GCN improves electrical conductivity and reduces the electron-transfer resistance of metal oxide and thus promotes the formation of Mn atoms and oxygen atoms into a covalent coordination bond or a hydrogen bond, such as Mn-OH.

Three fitted absorption bands can be observed in the N 1s spectrum in Figure S4. The peak around 398.6 eV resulted from N species in the C=N-C unit, while that at ca. 399.5 eV was assigned to the N-H groups in PDA and melamine sponge [11,26] and that around 400.2 eV to the N-(C)_3_ in GCN [2,27]. For MS/PDA/GCN/MnOx and MS/PDA/MnOx/GCN, the peak around 404.2 eV ascribed to π electron cloud density was caused by doped GCN [25].

### 3.4. Reaction Mechanism

**Figure 6:**
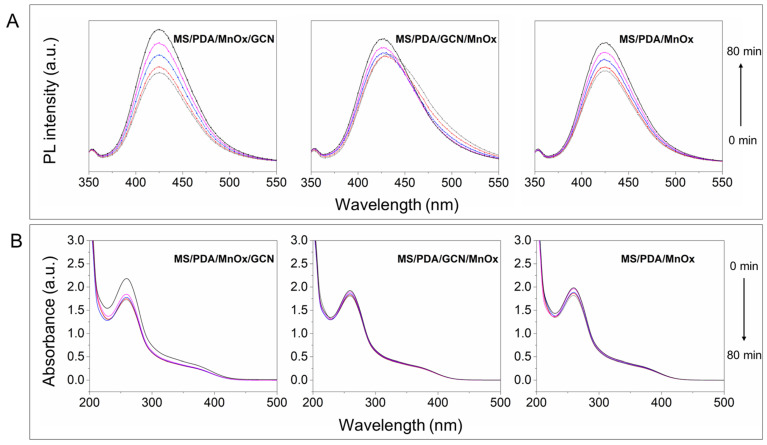
PL spectra of TA solution (**A**) and absorption spectra of NBT solution (**B**) with the catalysts MS/PDA/MnOx/GCN, MS/PDA/GCN/MnOx and MS/PDA/MnOx under xenon light irradiation.

**Figure 7:**
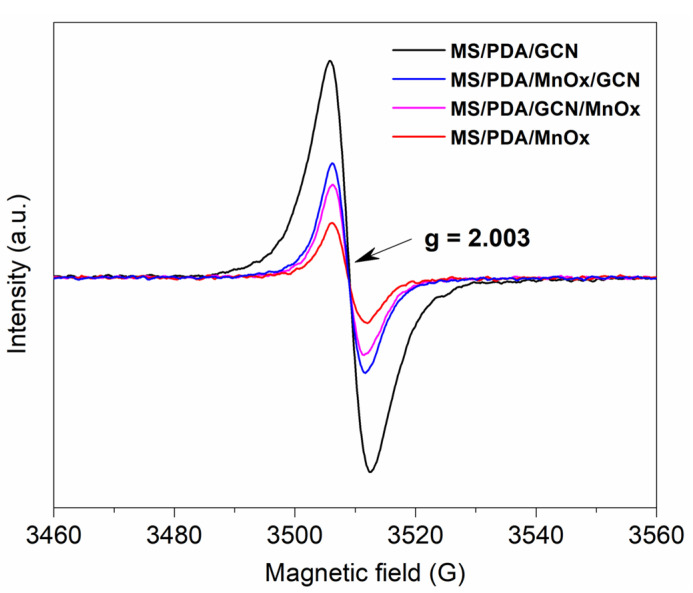
ESR test for the catalysts MS/PDA/GCN, MS/PDA/MnOx/GCN, MS/PDA/GCN/MnOx and MS/PDA/MnOx.

The photo-thermal-induced ROS were tested to further explore the reaction mechanism on the catalyst surface. The PL detection method was employed to confirm the generation of hydroxide radical with p-Phthalic acid (TA) as indicator. As shown in Figure 6A, the oxidative product TAOH had a sole fluorescence response at 425 nm [28]. It can be clearly found that the PL strength on the three Mn supported catalysts was significantly improved as the irradiation time increased, indicating that hydroxide was produced during the reaction. The formation of ·O^2^^−^ radical was also tested by nitro blue tetrazolium (NBT) as the indicator [29]. As shown in Figure 6B, the concentration of NBT over the catalysts MS/PDA/GCN/MnOx and MS/PDA/MnOx did not decrease significantly, meaning that O_2_ molecules were not activated on both catalysts. However, the generation of O^2^^−^ radical on MS/PDA/MnOx/GCN was obvious. The result implies that the outermost layer of GCN provides favorable conditions for the adsorption and activation of O_2_ molecules on the reaction interface, which was also confirmed by the ESR result for oxygen vacancy determination. As shown in Figure 7, all samples showed a pair of steep peaks with a symmetric distribution in accordance with g = 2.002, an indication of electron trapping at oxygen vacancies [30]. It can be concluded from the ESR result of the catalyst MS/PDA/GCN that the addition of GCN provides a large number of oxygen vacancies for the catalyst system. Additionally, the catalysts MS/PDA/MnOx/GCN had more oxygen vacancies than MS/PDA/GCN/MnOx and MS/PDA/MnOx, as the loaded GCN on the outermost layer was partially retained. The formation of sufficient oxygen vacancies promoted the absorption and activation of O_2_ atoms [31], leading to the formation of more ·O^2^^−^ radicals over MS/PDA/MnOx/GCN.

**Figure 8:**
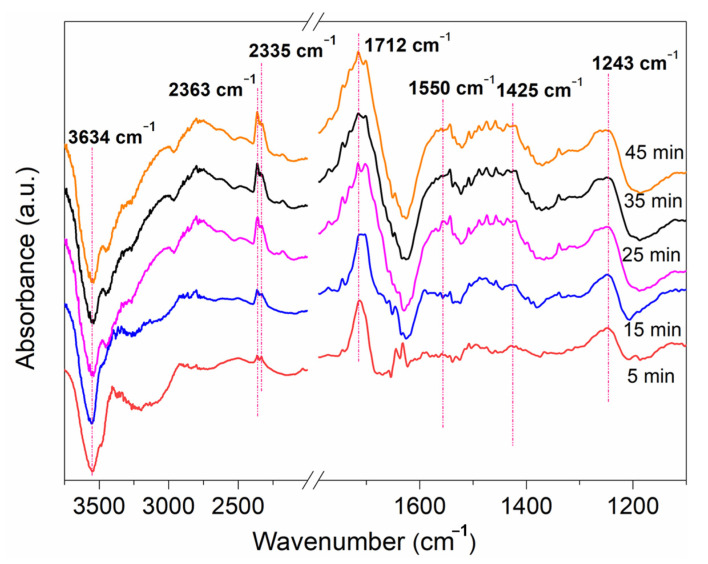
In situ DRIFTS of HCHO reaction on the catalyst MS/PDA/MnOx/GCN under xenon light irradiation.

In situ DRIFTS measurements were also conducted to obtain insight into the HCHO degradation mechanism. As shown in Figure 8, several vibration peaks were identified at approximately 3634, 2363, 2335, 1712, 1550, 1425 and 1243 cm^−1^. It can be found that the peak at 1425 cm^−1^ represented the vibrations of dioxymethylene (DOM) [32,33]. Additionally, another group peak at 1712 and 1550 cm^−1^ represented formate (HCOO^−^) species [32,33]. These are the primary degradation products of HCHCO oxidation. With the extension of reaction time, their corresponding vibration peaks gradually increased, indicating the absorption and accumulation of HCHO. The shoulder peaks at 2363 and 2335 cm^−1^ corresponded to CO_2_ adsorption with a corresponding vibration peak at 1243 cm^−1^ (assigned to the CO_3_^2−^ oxidized from HCOO^−^) [9,34], implying the final mineralization of HCHO. There was also a very strong negative peak at 3634 cm^−1^, which was assigned to hydroxyl vibration peak, representing a large consumption of Mn-OH during HCHO oxidation [35,36].

**Figure 9:**
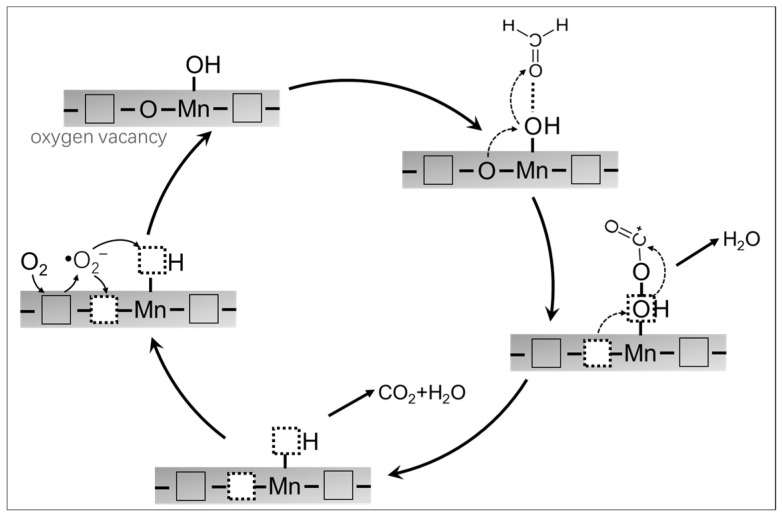
Probable degradation mechanism of HCHO over catalyst MS/PDA/MnOx/GCN.

Based on all the above, a plausible mechanism for HCHO oxidation over MS/PDA/MnOx/GCN is proposed as Figure 9. HCHO molecules were firstly adsorbed onto Mn-OH groups through hydrogen bonding [37,38], further utilizing adjacent Mn-O to form CO_2_ and H_2_O. Concurrently, O_2_ molecules absorbed onto conterminal oxygen vacancies to activate O^2^^−^ and were involved in the formation of CO_2_ and the replenishment of Mn-O.

### 3.5. Reusability

**Figure 10:**
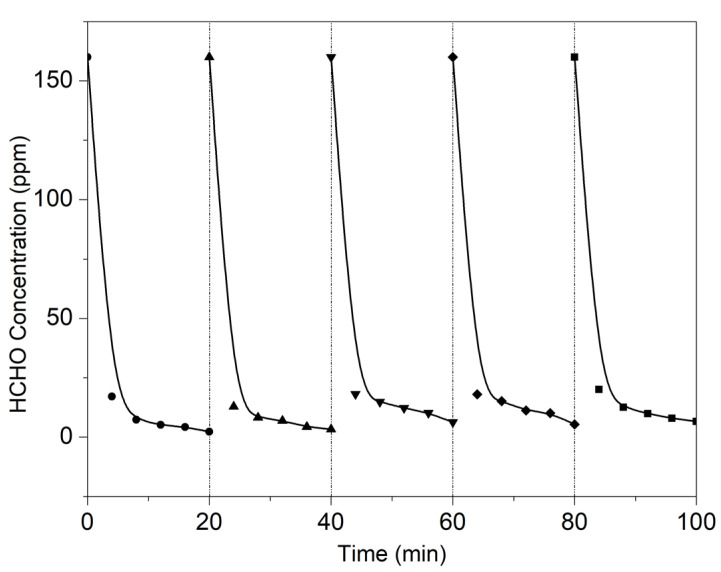
Cyclic performance test for HCHO removal.

The cyclic performance of catalysts is very important for photocatalytic materials, which can be used to estimate whether the catalyst is easy to deactivate. The deactivation of a catalyst always arises due to the loss of intrinsic (per-site) activity, or a decrease in the number of active sites, or increasingly restricted access to the active sites [39]. Based on the above in situ DRIFTS analysis and mechanism elaboration results, we can speculate that the most likely cause of deactivation over this catalyst was only due to the accumulation of residue at the active site. As shown in Figure 10, HCHO was purified by the MS/PDA/MnOx/GCN catalyst five times. The results showed that the purification effect of HCHO was basically the same as that purified for the first time. Although it would be wrong to repeat the experiment to measure the stability of the catalyst at 100% conversion without studying under kinetically controlled conditions [39], we could not measure stability at the 50% or 90% conversion rates because the high sensitivity of this catalyst could achieve a 100% conversion rate of HCHO in a very short time. However, it is worth noting that the MS/PDA/MnOx/GCN catalyst exhibited excellent reuse performance for at least five tests, which indicates the potential to be developed as a commercial catalyst against indoor HCHO removal.

## 4. Conclusions

To sum up, we designed an integral HCHO photocatalyst characterized by impressive light-to-heat conversion property and excellent reusability. The concentration of HCHO dropped from 160 ppm to 46 ppm within 20 min under the irradiation of visible light. The addition of GCN promoted the formation of oxygen vacancy, thus facilitating the activation of surface reactive oxygen species and maintaining the efficient oxidation of HCHO under a spontaneously rising surface temperature. The association of GCN with MnOx over common three-dimensional sponge materials offers great potential for commercial catalyst development aimed at indoor HCHO control without using noble metals, which also makes a significant contribution to the literature regarding catalysts designed to reduce environmental air pollution.

## Data Availability

Not applicable.

## Supplementary Materials

The following supporting information can be downloaded at: https://www.mdpi.com/article/10.3390/molecules27165216/s1, Figure S1: UV–Vis-NIR DRS profiles of catalyst MS/PDA, MS/PDA/MnOx, MS/PDA/GCN/MnOx, and MS/PDA/MnOxGCN; Figure S2: Changes in surface temperature of catalyst MS/PDA, MS/PDA/MnOx, MS/PDA/GCN/MnOx, and MS/PDA/MnOxGCN over visible light irradiation; Figure S3: The thermal imagery of catalyst MS/PDA, MS/PDA/MnOx, MS/PDA/GCN/MnOx, and MS/PDA/MnOxGCN over visible light irradiation; Figure S4: N 1s XPS spectra of catalyst MS/PDA/MnOx, MS/PDA/GCN/MnOx and MS/PDA/MnOx/GCN; Figure S5: Photos of the experiment process.

## Sample Availability

Samples mentioned in paper are available from the authors.
