# Supplementary material for: A Three-Dimensional Melamine Sponge Modified with MnOx Mixed Graphitic Carbon Nitride for Photothermal Catalysis of Formaldehyde
Source: Molecules. 2022 Aug 16;27(16):5216. doi: 10.3390/molecules27165216 (PMC9416345; doi:10.3390/molecules27165216)
Supplement: Supplementary file 1 [file molecules-27-05216-s001.zip › molecules-1850198-supplementary.pdf]

A Three-dimensional Melamine Sponge Modified with MnOx Mixed Graphitic Carbon Nitride for Photothermal Catalysis of Formaldehyde

Rongyang Yin, Pengfei Sun*, Lujun Cheng, Tingting Liu, Baocheng Zhou, and Xiaoping Dong

Department of Chemistry, Key Laboratory of Surface & Interface Science of Polymer Materials of Zhejiang Province, Zhejiang Sci-Tech University, 928 Second Avenue, Xiasha Higher Education Zone, Hangzhou 310018, China

**Corresponding author: sunpf@zju.edu.cn (P. Sun)*

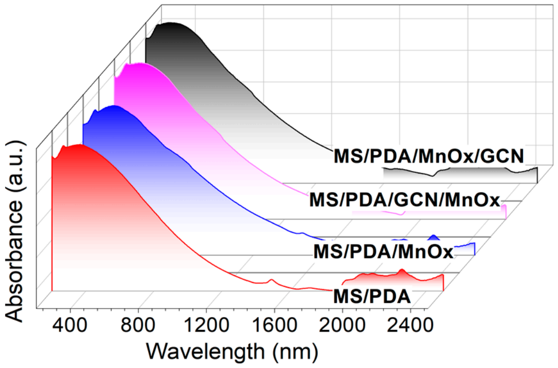

Figure S1. UV–Vis-NIR DRS profiles of catalyst MS/PDA, MS/PDA/MnOx, MS/PDA/GCN/MnOx, and MS/PDA/MnOxGCN.

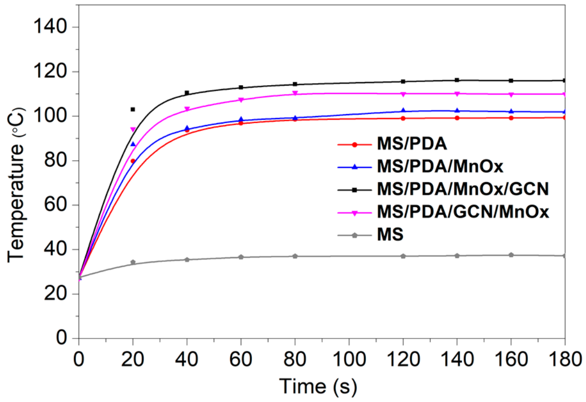

Figure S2. Changes in surface temperature of catalyst MS/PDA, MS/PDA/MnOx, MS/PDA/GCN/MnOx, and MS/PDA/MnOxGCN over visible light irradiation.

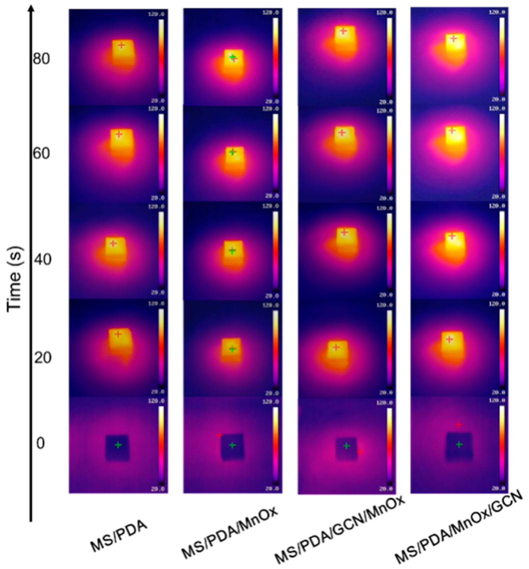

Figure S3. The thermal imagery of catalyst MS/PDA, MS/PDA/MnOx, MS/PDA/GCN/MnOx, and MS/PDA/MnOxGCN over visible light irradiation.

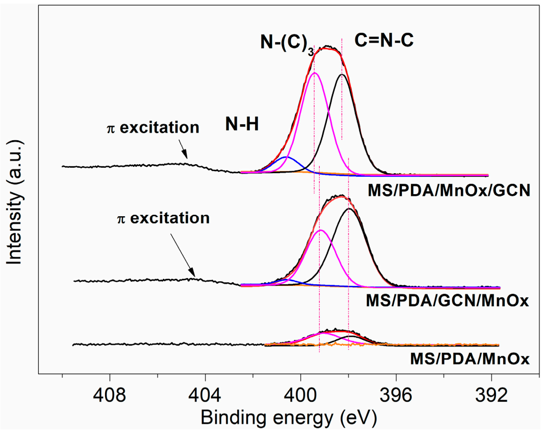

Figure S4. N 1s XPS spectra of catalyst MS/PDA/MnOx, MS/PDA/GCN/MnOx and MS/PDA/MnOx/GCN.

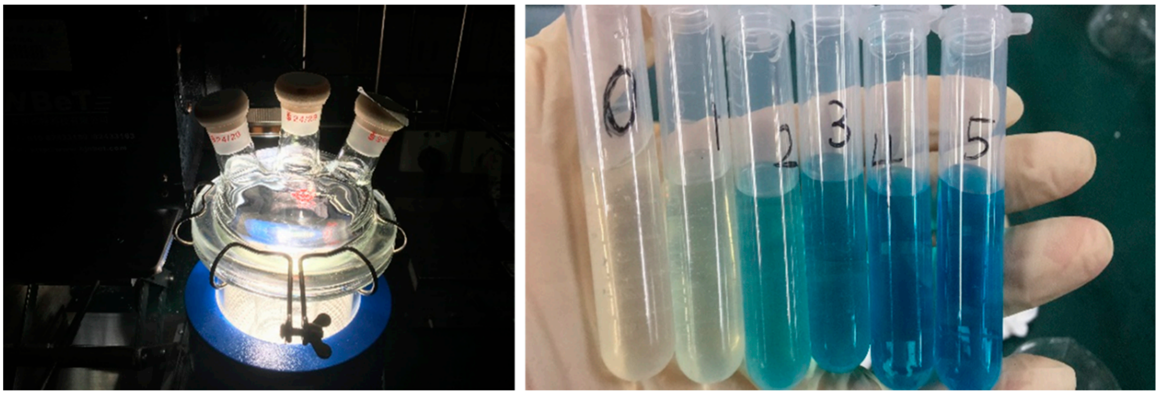

Figure S5. Photos of the experiment process.
